# Diagnostic value of the lymphocyte transformation test for non-IgE mediated food allergy

**DOI:** 10.1186/2045-7022-5-S3-P31

**Published:** 2015-03-30

**Authors:** Ivana Setinova, Marie Havranova, Eva Dankova, Marcela Vorlickova

**Affiliations:** 1 Immunia, Prague, Czech Republic

## Background

The aim of our study was to verify the diagnostic value of lymphocyte transformation test (LTT) for non-IgE mediated food allergy.

## Methods

We investigated 3 groups of patients for identifying the food hypersensitivity.

1. Group - 60 patients with symptoms of irritable bowel syndrome (IBS) without a diagnosis of organic gastrointestinal disease. 2. Group - 19 patients with atopic dermatitis, polysenzitization to inhalant, not to food allergens. 3. Group - 6 infants (3-12 months old) with intestinal symptoms (bloody stool and abdominal distention) and positive elimination diet with cow's milk. Control group -10 healthy non-atopic patients.

We performed skin prick test (SPT) and the flow-cytometric basophil activation test(BAT) with commercial extracts of wheat, rye flour, egg, milk and soy allergens Alyostal (Stallergenes) in all patients. Serum levels of milk, casein, wheat, rye flour, egg and soy specific IgE were measured by Immulite. The lymphocyte transformation test was performed with the same food allergens. Open food challenge test (OFC) was completed after 14 days of elimination diet.

## Results

All patients had negative SPT, sIgE and BAT with milk, casein, wheat, rye, egg and soy allergens. They had negative celiac disease serology. In 1.group 42 patients (70%) had positive LTT for wheat flour, none of them for other allergens. Open food challenge test by ingestion of daily serving cooked wheat pasta were positive in all 42 positive patients. 18 patients (30%) had negative LTT for all allergens. In 2.group 15 patients had positive LTT for wheat flour, 2 for milk, 1 for soy allergens Elimination diet of wheat or milk or soy improved eczematic manifestations. In 3.group all 6 infants had positive LTT to cow/s milk and casein allergens. They had positive OFC test with cow/s milk after elimination diet.

Patients of control group were negative in all vitro tests.

## Conclusion

In our study the lymphocyte transformation test were useful diagnostic tools for non-IgE mediated food allergy.

